# Trends in Varying Modes of Hospital Arrival: What Determines the Ideal Mode of Transportation?

**DOI:** 10.7759/cureus.98183

**Published:** 2025-11-30

**Authors:** Andrew W Gelis, Austin Henken-Siefken, Andrew McCague

**Affiliations:** 1 Research, Rocky Vista University College of Osteopathic Medicine, Englewood, USA; 2 Surgery, Desert Regional Medical Center, Palm Springs, USA; 3 Trauma and Acute Care Surgery, Desert Regional Medical Center, Palm Springs, USA

**Keywords:** ambulance transport, emergency medicine and trauma, general trauma surgery, surgery general, total mortality rate, trauma center, trend analysis

## Abstract

Introduction

Modes of transportation to hospitals can significantly alter the morbidity and mortality rates of medical emergencies. Identifying different modes of transportation as risk factors for mortality in emergency room (ER) visits can enhance understanding of safety. This study aimed to investigate the relationship between modes of transportation and mortality and to see how trends in various patient factors change over time.

Methods

A retrospective analysis was conducted utilizing data from the National Trauma Data Bank (NTDB) from 2012 to 2022. The study consisted of patients who arrived at an emergency room. Data regarding different modes of arrival and mortality outcome were queried, along with additional variables including patient age, sex, length of stay (LOS), injury severity score (ISS), and hospital discharge disposition. Chi-square analysis was performed to assess the independent association between these variables.

Results

A total of 11,716,771 patients were included. The mode of transportation among those who expired was as follows: 75.64% (215,059 patients) used ground ambulance, 20.66% (58,746 patients) used helicopter emergency medical services (HEMS), 2.63% (7,488 patients) used private/public vehicle/walk-ins, 0.71% (2,007 patients) used fixed-wing ambulance, and 0.36% (1,035 patients) used police. After adjustment for covariates, the analysis revealed a statistically significant (p<0.001) association across all years. The transportation mode with the highest percentage of total deaths across all years was helicopter ambulance (6.84%), followed by fixed-wing ambulance (4.28%) and ground ambulance (2.46%). Mortality rate and LOS increased across all modes of transportation from 2012 to 2022, except for police transports, which experienced decreased rates.

Conclusion

These data suggest that certain modes of transportation may face increased mortality risk, with the highest percentage of deaths from helicopter ambulance. With increased usage of certain modes of transportation over time, it allows us to question whether such transportation modes are justified by risk and cost. Further research is warranted to elucidate the underlying mechanisms contributing to these disparities.

## Introduction

Emergency departments (EDs) are often the first point of contact many people have with the healthcare system. With increasing difficulty in finding primary care physicians (PCPs), many are often forced to use EDs for routine medical care. With many EDs often filled to capacity at all hours of the day, hospitals struggle to manage the never-ending flux of patients from all over their region. Routinely, we imagine transportation to EDs via ground ambulance, but advancements in technology and emergency medical transportation have created new methods such as helicopter emergency medical services (HEMS) and fixed-wing ambulances, options that are often useful when faster transport is required. Despite numerous modes of transport available to reach hospitals, a fast emergency medical service (EMS) response is critical to help improve clinical outcomes in severely injured patients, particularly for those who lack advanced EMS [1,2]. Depending on the method and severity of injury, delays in transport to trauma centers are often associated with higher mortality rates in adult patients [3,4]. As a result, some studies show that HEMS can increase the mortality benefit in adult patients [5,6]. Nevertheless, because of the varying availability of such services and types of trauma cases encountered throughout, there is still conflicting data on the best prehospital care management as a whole [7].

The objective of our study was to understand how rates of mortality vary across different modes of transportation to hospitals, while subsequently analyzing trends in utilization and patient demographics over time. We also aimed to understand the trends of varying modes of transportation over time and understand how they affect other variables such as hospital length of stay (LOS), injury severity score (ISS), sex, and average age of patients who use such transport services. The study analyzed data from 2012 to 2022 and identified different modes of transportation as risk factors for mortality to determine which modes are the safest. We hypothesized that ground ambulances would be associated with higher rates of mortality compared to other modes due to the increased usage of such methods of transportation in general emergency services.

## Materials and methods

We performed a retrospective analysis of the American College of Surgeons National Trauma Data Bank (NTDB) from 2012 to 2022. For each year, SAS files categorized as trauma, transport, demographics, abbreviated injury scale (AIS), and discharge were utilized to cumulate the necessary demographics and transportation mode used and cross-referenced with the participant use file (PUF) dictionary to correctly categorize each mode of transportation with their referred numerical value. All trauma patients were de-identified from across the United States, and patient consent was waived as no identifiable information was used or available. The primary outcome of the study was to see how rates of mortality differed when compared across numerous modes of transportation, including ground ambulance, HEMS, fixed-wing ambulance, private/public vehicle/walk-in, and police.

Patients of all ages were included in this study. Modes of transportation and mortality outcome were listed as categorical variables. Other factors, such as the injury severity score (ISS), average age, average LOS, total number of cases (N), and total number of expirations, were listed as continuous variables. Incidence was kept at two decimal points rather than rounded to the nearest whole number to better show an accurate representation of the entire dataset, given its large size. All statistical analyses were performed using the Statistical Package for the Social Sciences (SPSS) software version 30, developed by IBM in Armonk, New York, USA. No artificial intelligence (AI) was used in this study.

Chi-square analyses, one-way ANOVAs, and various frequency tables were calculated to assess the independent and statistically significant association between transportation mode and mortality, LOS, average age, sex, and ISS. Subsequent analysis suggested an effect modification by year, allowing us to trend these variables to see how they changed over a 10-year time period. By analyzing data across each year, we were able to create frequency tables to better assist with our analysis. Various other factors regarding patient outcomes, including but not limited to patients leaving against medical advice (AMA) and patient transfers, were excluded as they were not related to the purpose of this study.

## Results

Through the NTDB database, 15,524,589 patients were initially analyzed, of which 11,716,771 had a classified mode of transportation and were hence included in the study. The remaining excluded cases (3,807,818 patients) either had an unknown, unrecorded, or “other” mode of transportation, and hence did not meet our inclusion criteria. Among the included cases (Table 1), 2.43% of all patients (284,335 patients) expired per their hospital discharge disposition. Among those patients who expired, 75.64% (215,059 patients) used ground ambulance, 20.66% (58,746 patients) used HEMS, 2.63% (7,488 patients) used private/public vehicle/walk-ins, 0.71% (2,007 patients) used fixed-wing ambulance, and 0.36% (1,035 patients) used police as modes of transportation to hospitals. After adjustment for relevant covariates, the analysis revealed that there was a statistically significant (p<0.001) association between all modes of transport and hospital discharge disposition (expiration), ISS, average age, sex, and LOS from 2012 to 2022.

**Table 1:** Demographic Table SD: standard deviation, ISS: injury severity score, LOS: length of stay, ANOVA: analysis of variance

Mode of transportation/analysis	N (count)	Age (mean)	SD	Sex (majority)	ISS (average)	LOS (days) (mean)	Expired (% mortality)
Ground ambulance	8,761,871	61.43	20.42	Male	8.96	5.85	215,059 (2.46%)
Helicopter ambulance	859,426	53.67	20.08	Male	14.49	8.86	58,746 (6.84%)
Fixed-wing ambulance	46,952	56.19	20.67	Male	12.83	9.09	2,007 (4.28%)
Private/public vehicle/walk-ins	2,004,559	58.32	21.51	Male	5.76	3.84	7,488 (0.37%)
Police	43,963	43.14	16.47	Male	8.88	5.36	1,035 (2.35%)
Chi-square P-value	-	-	-	<0.001	<0.001	<0.001	<0.001
One-way ANOVA	-	<0.001	-	-	-	-	-

When trending across each year, the number of total annual deaths across all modes of transport, a sum of datapoints from Tables 2-6, were as follows: 21,341 deaths in 2012; 21,020 deaths in 2013; 21,406 deaths in 2014; 22,878 deaths in 2015; 23,182 deaths in 2016; 25,914 deaths in 2017; 26,185 deaths in 2018; 27,067 deaths in 2019; 28,993 deaths in 2020; 32,987 deaths in 2021; and 33,362 deaths in 2022. When comparing the relative number of deaths across all years (2012-2022) per mode of transportation, HEMS (Table 4) had the highest percentage of deaths within its own category at 6.84% (58,746 patients), followed by 4.28% (2,007 patients) for fixed-wing (Table 2), 2.46% (215,059 patients) for ground ambulance (Table 3), 2.35% (1,035 patients) for police (Table 5) and 0.37% (7,488 patients) for private/public vehicle/walk-ins (Table 6).

**Table 2:** Statistical Annual Trends Across Fixed-Wing Ambulances SD: standard deviation, LOS: length of stay

Year	N	Incidence (N)	Age (mean)	SD	LOS (days)	Mortality (% of cases)	Mortality rate (per 1,000)
2012	4,587	97.7	54.05	20.85	9.08	159 (3.47%)	34.66
2013	5,140	109.47	55.84	21.07	8.73	150 (2.92%)	29.18
2014	5,399	114.99	57.05	20.94	8.36	164 (3.04%)	30.38
2015	5,444	115.95	56.46	20.81	8.82	157 (2.88%)	28.84
2016	5,385	114.69	55.32	20.31	8.41	174 (3.23%)	32.31
2017	4,117	87.69	55.85	20.51	9.06	193 (4.69%)	46.88
2018	3,456	73.61	56.45	20.32	8.87	145 (4.20%)	41.96
2019	3,245	69.11	57.23	20.24	9.48	279 (8.60%)	85.98
2020	3,325	70.82	56.24	20.68	9.35	172 (5.17%)	51.73
2021	3,341	71.16	56.62	20.99	9.67	245 (7.33%)	73.33
2022	3,513	74.82	56.94	20.66	10.12	169 (4.81%)	48.11
R-value	-0.81	-0.81	0.59	-	0.77	0.48	0.69
Chi-square P-value	-	-	<0.001	-	<0.001	<0.001	-

**Table 3:** Statistical Annual Trends Across Ground Ambulances SD: standard deviation, LOS: length of stay

Year	N	Incidence (N)	Age (mean)	SD	LOS (days)	Mortality (% of cases)	Mortality rate (per 1,000)
2012	664,596	75.85	58.5	21.33	5.48	15175 (2.28%)	22.83
2013	681,091	77.73	59.39	21.10	5.39	15298 (2.25%)	22.46
2014	708,741	80.89	60.06	20.90	5.43	15704 (2.22%)	22.16
2015	780,175	89.04	60.5	20.79	5.51	17195 (2.20%)	22.04
2016	835,359	95.34	60.87	20.73	5.12	17353 (2.08%)	20.77
2017	746,955	85.25	61.44	20.45	5.87	19556 (2.62%)	26.18
2018	789,263	90.08	62.28	20.10	5.83	20109 (2.55%)	25.48
2019	829,142	94.63070159	62.81	19.88	5.85	20458 (2.47%)	24.67
2020	859,688	98.11694329	62.47	20.01	5.93	22434 (2.61%)	26.1
2021	923,105	105.354781	63.18	19.83	6.77	25410 (2.75%)	27.53
2022	943,756	107.71	64.22	19.46	7.11	26367 (2.79%)	27.94
R-value	0.94	0.94	0.99	-	0.83	0.97	0.84
Chi-square P-value	-	-	<0.001	-	<0.001	<0.001	-

**Table 4:** Statistical Annual Trends Across Helicopter Ambulances SD: standard deviation, LOS: length of stay

Year	N	Incidence (N)	Age (mean)	SD	LOS (days)	Mortality (% of cases)	Mortality rate (per 1,000)
2012	83,894	97.61	51.56	20.01	8.61	5351 (6.38%)	63.78
2013	77,943	90.69	52.25	19.92	8.39	4926 (6.32%)	63.2
2014	80,178	93.29	52.79	20.03	8.31	4880 (6.09%)	60.87
2015	80,460	93.62	53.01	20.10	8.46	4808 (5.98%)	59.7564007
2016	78,743	91.62	53.15	20.16	7.98	4980 (6.32%)	63.24
2017	74,698	86.92	53.88	20.09	8.92	5426 (7.26%)	72.64
2018	70,331	81.83	54.5	20.00	8.87	5143 (7.31%)	73.13
2019	72,437	84.29	55.07	20.03	8.9	5554 (7.67%)	76.67
2020	76,740	89.29	54.24	20.15	9.08	5510 (7.18%)	71.8
2021	81,404	94.72	55.33	20.20	9.86	6286 (7.72%)	77.22
2022	82,598	96.11	56.56	20.23	10.07	5882 (7.12%)	71.21
R-value	-0.19	-0.19	0.96	-	0.82	0.77	0.8
Chi-square P-value	-	-	<0.001	-	<0.001	<0.001	-

**Table 5:** Statistical Annual Trends Across Police SD: standard deviation, LOS: length of stay

Year	N	Incidence (N)	Age (mean)	SD	LOS (days)	Mortality (% of cases)	Mortality rate (per 1,000)
2012	5,059	115.07	47.1	18.95	6.3	136 (2.69%)	26.88
2013	5,320	121.01	46.14	18.48	6.17	108 (2.03%)	20.3
2014	5,555	126.36	47.37	18.62	5.82	115 (2.07%)	20.7
2015	4,011	91.24	43.77	17.11	5.32	89 (2.22%)	22.19
2016	3,282	74.65	41.33	15.90	3.81	39 (1.19%)	11.88
2017	3,340	75.97	43.85	16.63	5.36	98 (2.93%)	29.34
2018	3,634	82.66	44.06	16.95	5.57	112 (3.08%)	30.82
2019	3,067	69.76	40.5	14.76	4.59	65 (2.12%)	21.19
2020	3,358	76.38	39.86	14.73	4.85	95 (2.83%)	28.29
2021	3,597	81.82	40.04	14.51	5.44	108 (3.00%)	30.03
2022	3,740	85.07	40.47	14.49	5.67	70 (1.87%)	18.72
R-value	-0.73	-0.73	-0.88	-	-0.36	-0.4	0.19
Chi-square P-value	-	-	<0.001	-	<0.001	<0.001	-

**Table 6:** Statistical Annual Trends Across Private/Public Vehicle/Walk-Ins SD: standard deviation, LOS: length of stay

Year	N	Incidence (N)	Age (mean)	SD	LOS (days)	Mortality (% of cases)	Mortality rate (per 1,000)
2012	167,575	83.6	56.09	22.13	3.54	520 (0.31%)	3.1
2013	170,290	84.95	56.67	21.88	3.54	538 (0.32%)	3.16
2014	181,685	90.64	57.19	21.73	3.6	543 (0.30%)	2.99
2015	199,672	99.61	57.78	21.62	3.58	629 (0.32%)	3.15
2016	220,039	109.77	58.11	21.65	3.24	636 (0.29%)	2.89
2017	156,903	78.27	58.88	21.42	4	641 (0.41%)	4.09
2018	165,174	82.4	59.66	21.03	3.93	676 (0.41%)	4.09
2019	178,516	89.06	59.88	21.02	3.91	711(0.40%)	3.98
2020	183,980	91.78	57.93	21.47	3.82	782 (0.43%)	4.25
2021	189,974	94.77	59.1	21.44	4.45	938 (0.49%)	4.94
2022	190,751	95.16	60.25	21.22	4.67	874 (0.46%)	4.58
R-value	0.18	0.18	0.86	-	0.81	0.95	0.9
Chi-square P-value	-	-	<0.001	-	<0.001	<0.001	-

When trending the average age of patients from 2012 to 2022 per mode of transportation (Table 1), the average age of patients using ground ambulances was 61.43 years old, with an average LOS of 5.85 days and ISS of 8.96. The average age of patients using HEMS (Table 1) was 53.67 years old, with an average LOS of 8.86 days and ISS of 14.49. The average age of patients using fixed-wing ambulances (Table 1) was 56.19 years old, with an average LOS of 9.09 days and ISS of 12.83. The average age of patients using private/public vehicles/walk-ins (Table 1) was 58.32 years old, with an average LOS of 3.84 days and ISS of 5.76. The average age of patients brought in by police (Table 1) was 43.14 years old, with an average LOS of 5.36 days and ISS of 8.88.

**Figure 1:**
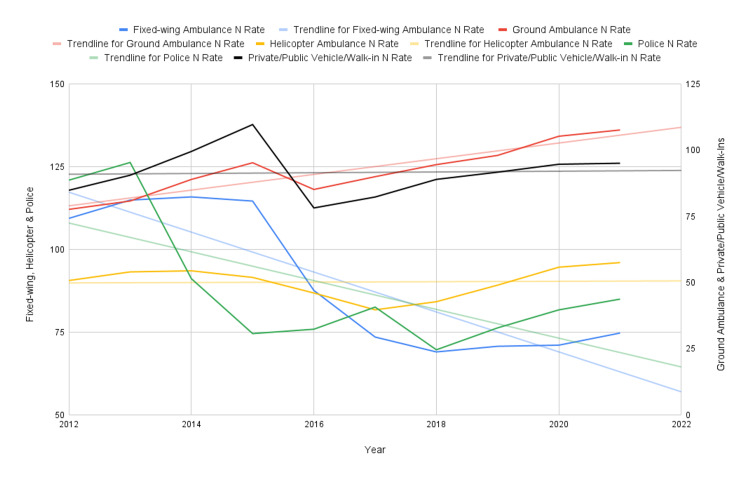
Trends in Prevalence (N) Rates for Transportation Modes (2012-2022) Figure 1 shows how the N value per 1,000 for each mode of transportation was trended from 2012 to 2022. The left Y-axis measures values for fixed-wing, helicopter, and police, while the right Y-axis shows the values for ground ambulances and private/public vehicle/walk-ins. Each dataset also has a trendline associated with it to better show the overall direction of the rate.

**Figure 2:**
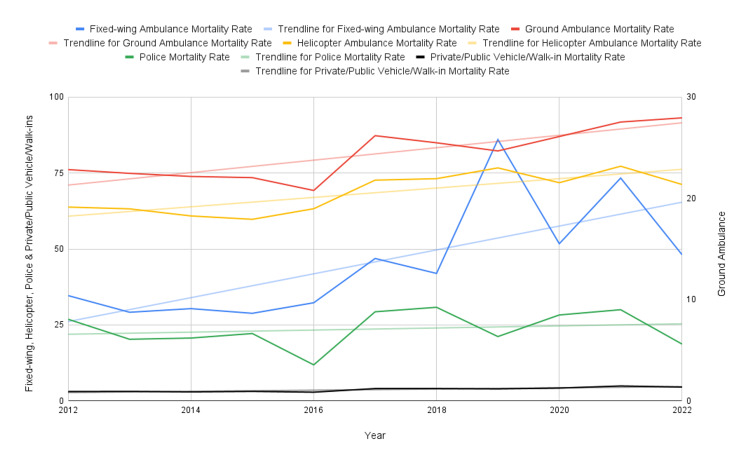
Trends in Mortality Rates for Transportation Modes (2012-2022) Figure 2 shows how the mortality rate (per 1,000) for each mode of transportation was trended from 2012 to 2022. The left Y-axis measures values for fixed-wing, helicopter, police, and private/public vehicle/walk-ins, while the right Y-axis shows the values for ground ambulances. Each dataset also has a trendline associated with it to better show the overall direction of the rate.

Regarding total usage of each mode of transportation from 2012 to 2022 (Figure 1), the number of fixed-wing ambulances (Table 2) decreased (R-value of -0.81), with a relatively stable mortality count (R-value of 0.48). Average LOS (R-value of 0.77) and average age (R-value of 0.59) for fixed-wing ambulances did not vary by large degrees. Ground ambulance usage (Table 3) increased (R-value of 0.94), as well as average age (R-value of 0.99) and mortality count (R-value of 0.97), with otherwise constant LOS (R-value of 0.83). HEMS usage (Table 4) saw a slight decrease in usage over the time period (R-value of -0.19), with a slight increase in average age (R-value of 0.96), LOS (R-value of 0.81), and mortality count (R-value of 0.76). Police escorts (Table 5) saw a relatively larger decrease in usage over the years (R-value of -0.73), with a decreasing average age (R-value of -0.88), LOS (R-value of -0.37), and decreasing mortality count (R-value of -0.40). Finally, private/public vehicle/walk-ins (Table 6) saw a rather stable number of cases (R-value of 0.18), with slightly increasing mortality count (R-value of 0.95), average age (R-value of 0.86), and LOS (R-value of 0.81) across all years. The trends in mortality rates associated with each mode of transportation compared with each other from 2012 to 2022 can be seen in Figure 2.

## Discussion

Mortality rates can drastically change based on various factors such as ISS, average LOS, average age, sex, and mode of transportation to trauma centers. With emerging technologies providing faster and more efficient transportation to hospitals, it is necessary to consider which transportation modes would provide the best opportunities for each individual patient’s medical and financial needs. Naturally, a higher ISS often predicts a higher mortality rate, necessitating more advanced emergency transportation services such as HEMS. Additional factors that may require more advanced medical transport services include distance from the closest trauma center, necessity of tertiary care centers, availability of various modes of transport, and rate of patient decline. Given the raw number of patients included in this study, it provides a unique opportunity over a large timespan to analyze which modes of transportation are most effective for patients of all ages and backgrounds across the United States.

There have been multiple studies that often try to find the best type of transportation for trauma patients [8]. Papers by Bulger et al. showed that factors such as longer distances or greater time to care with higher ISS resulted in equivalence of survival [9]. In low ISS cases, such as a minor laceration requiring stitches in the emergency room (ER), many patients may often opt to drive themselves to hospitals due to faster timing and decreased financial burden from private ambulances. There have been numerous studies that state that, due to various reasons, patients transported by private vehicles are less likely to die compared to ground ambulances [10-12]. Nevertheless, medicolegal fears may hinder private vehicle usage due to liability concerns. It is thus important to consider varying factors in emergency settings when determining what the most appropriate mode of transportation is for each patient.

Our study revealed that HEMS transportations had the highest average ISS from 2012 to 2022 compared to other modes of transportation, such as ground ambulance. The higher ISS signified a more acute and traumatic case, which can explain the higher need for advanced life support (ALS) as opposed to private vehicle transportation. This statistic further highlights the benefits of HEMS, which entails a higher level of training among medical staff, among other benefits as highlighted in other literature [13-15].

Our data showed a 6.84% mortality across all years in patients using HEMS (Table 1), which had the highest percentage of mortality per mode of transportation. Nevertheless, with each subsequent year, the total number of deaths across all modes of transportation also increased from 2012 to 2022. With the highest ISS at 14.49 (Table 1), HEMS patients’ more life-threatening injuries could explain why they were more likely to die despite having highly trained medical personnel. The high mortality rate (6.84%) is likely a reflection of patients’ pre-existing critical condition rather than an indictment of HEMS care. On the contrary, private/public vehicle/walk-ins had both the lowest ISS (5.76) and percentage of mortality (0.37%) among all groups from 2012 to 2022 (Table 1), which shows a direct correlation between these variables. With less acute injuries and needs for medical care, we expect patients to have a higher survival rate and hence a lower need for more advanced modes of transportation such as HEMS.

When comparing average LOS across different modes of transportation, the results showed that the highest average LOS was among fixed-wing (average LOS: 9.09 days) and HEMS (average LOS: 8.86 days) patients from 2012 to 2022 (Table 1), and the lowest average LOS was among private/public vehicle/walk-ins (average LOS: 3.84 days). All modes of transportation over this time period had an average LOS R-value between 0.77 and 0.83, except for police, which was at -0.36. For modes of transportation aside from police, this indicates a strong positive linear relationship, suggesting that average LOS for patients who use ground ambulance, fixed-wing ambulance, HEMS, and private/public vehicle/walk-ins are experiencing more days in the hospital over the 10-year time period. On the contrary, we saw a weaker yet negative relationship between LOS and police transportation (R-value of -0.36), as seen in Table 5. With a decrease in the total number of police transportations from 2012 to 2022 (number of cases, R-value of -0.73), this suggests that decreased usage in this mode of transportation may have directly led to a lower LOS. Police officers do not receive the same extent of medical training as ambulance EMS, which could explain why a decrease in police transports is associated with a decrease in mortality. As a result, additional medical training for police officers may further decrease mortality rates associated with police transports. We also saw a strong decrease in the average age of police transport patients (R-value of -0.88) from 2012 to 2022. With younger patients being incorporated more into police transports, this may also explain why the average LOS and mortality count are decreasing, as younger patients are often healthier than their older counterparts, leading to less time spent in hospitals. Additionally, younger patients typically have fewer comorbid conditions, which often delay recovery, decreasing average lengths of stay.

Fixed-wing ambulances saw an uptrending LOS (R-value of 0.77) despite a decrease in the number of cases (R-value of -0.81), as seen in Table 2. Being a costly and less available form of transportation, this may suggest a decrease in the number of trained personnel available to care for the few patients that require such a specialized form of transportation. An increasing LOS may suggest that higher costs are not justified for this mode of transportation. On the contrary, fixed-wing ambulances may still be useful at subsidized rates in highly remote areas such as Alaska, where large medical centers may be extremely far away. Nevertheless, fixed-wing ambulances require a runway with a large space and often a second form of medical transportation, such as ground ambulances, to enter the hospital. As a result, HEMS may be justified in such cases over fixed-wing ambulances, as it was shown to have around the same average LOS (8.86 days for HEMS versus 9.09 days for fixed-wing) despite a slightly higher mortality count (6.84% for HEMS versus 4.28% for fixed-wing ambulances). Additionally, HEMS can often land above trauma centers, not requiring a large landing space and a second mode of transportation to enter a medical facility, as required by many fixed-wing ambulances.

Ground ambulances saw a rising number of cases (R-value of 0.94) and a rising LOS (R-value of 0.83) from 2012 to 2022 (Table 3), suggesting that more ground ambulance use is increasing resource burdens on hospitals. With a rising average age among ambulance patients (R-value of 0.99) from 58.5 years old in 2012 to 64.22 years old in 2022, the average number of comorbidities and diseases associated with these patients rises, which could contribute to the increased usage and average LOS among ground ambulance patients. With the average age of ground ambulance patients in 2022 being almost the same age as that to qualify for Medicare (64.22 years old versus 65 years old, respectively), we may begin to question how many of these ambulance services are emergency necessities and how many are used for less emergent medical transportation with better insurance coverage.

With a slight decrease in the number of HEMS (Table 4) use from 2012 to 2022 (R-value of -0.19), we still saw a rise in the average LOS (R-value of 0.82) and average age (R-value of 0.96). With a naturally aging US population, increasing comorbidities and disease burdens in older patients may directly contribute to the increasing average LOS among HEMS patients. Nevertheless, this mode of transportation is extremely costly, and despite having an increased LOS, the average LOS of ground ambulances is much shorter, which may suggest the need to substitute more HEMS flights for ground ambulance transportation when there is availability and the ISS is not significantly high, among other variables.

Private/public vehicle/walk-ins (Table 6) saw a rise in average LOS (R-value of 0.81), as well as a rise in average age (R-value of 0.86). Nevertheless, we did not see a dramatic increase in the number of patients who use this mode of transportation from 2012 to 2022 (R-value of 0.18), suggesting that these increases in LOS may again be related to our aging US population. This may reflect the substitution patients employ of visiting ERs rather than PCPs for routine medical care due to the infrequency and unavailability of appointments in primary care. While we would have hoped to see a downtrend in private/public vehicle/walk-in usage for hospital visits, this may further highlight our ever-growing need for additional primary care physicians and services across the country to decrease the ER burden.

When comparing sexes between modes of transportation from 2012 to 2022 (Table 1), all groups had a majority of male patients. With the average male exhibiting higher rates of extreme and impulsive behaviors compared to female counterparts, this may suggest why men make up the majority of cases among the more specialized forms of medical transportation, such as HEMS, police escorts, and fixed-wing ambulances. Nevertheless, this is not always the case and can be subject to bias. Regarding private/public vehicle/walk-ins and ground ambulances usage, male patients may make up higher percentages due to decreased daily preventative care, among other tasks, when compared to their sex counterparts.

Depending on the type of transportation, financial burdens need to be considered as they can have a major effect on patient outcome, a variable that was not directly calculated in this study. Transportation distance, insurance coverage, and location of injury (rural versus urban) are additional factors that need to be considered in future studies in order to realistically understand which mode of transportation makes the most sense for any given patient. Given the large geographic variability in the United States, this poses a significant question when determining the ideal transportation mode for trauma patients.

A limitation of this study is the retrospective nature of the data analysis, which makes it more prone to selection and calculation bias. Despite being able to include large patient datasets in this study over a 10-year timespan, the retrospective nature limits our understanding of the current trends today. With the most recent dataset being from 2022, it raises a question as to whether these trends have changed today. With our datasets only including mortality rates from the hospital discharge disposition, we were unable to follow long-term trends of patients after discharge. This could play a major role in cases such as subsequent death or readmission for complications related to their original injury.

Nevertheless, our paper has provided highly important data trends and statistics regarding the usage of various modes of transportation from 2012 to 2022 and how other factors, such as ISS, average age, sex, and LOS, are affected. We were able to see how mortality has increased across all modes of transportation over time, except for police transports, which experienced a decrease (Figure 2). Fixed-wing ambulances and police transports experienced a decreasing rate in total usage over time, and ground ambulances experienced the largest increase in usage rate over time (Figure 1). With increasing rates of LOS and average age across most groups relative to the total population, except for police transports (Tables 2-6), these trends help us understand the further increasing burden on hospitals over the 10-year time period, which may only further worsen with our aging population. Despite an increasing usage of transportation services, we still saw a rising rate in mortality, highlighting concerns regarding continued high-quality care en route to hospitals.

## Conclusions

Our data showed an increasing usage and average ages of patients using ground ambulances and a decreased usage of fixed-wing ambulances from 2012 to 2022. HEMS saw a slight decrease in usage rate despite an increasing mortality count. Despite being a costly form of transportation, we need to question whether critically ill patients transported by HEMS had an improved survival compared to their outcome with alternative services. Ground ambulances increased usage and average age over time. Private/public vehicle/walk-ins saw a rather steady usage, with increasing average ages, mortality, and LOS, suggesting a possible direct reflection of our aging population. Police transportations saw a decreased average age, average LOS, and usage from 2012 to 2022, suggesting other methods of transportation may be used as alternatives. Additionally, male patients made up the majority of all patient groups in all modes of transportation.

With an overall rise in average LOS and average ages across most transportation modes, it remains difficult to discern if there is truly one superior mode of transportation that decreases mortality. Police transports were the only group to see a downtrend in mortality, with all other groups either remaining stable or rising. With continued analysis of other factors such as ISS, average age, sex, and LOS, understanding these disparities can inform targeted interventions and prevention strategies to mitigate the risk of death for all patient populations.
